# New Biomarkers in Acute Tubulointerstitial Nephritis: A Novel Approach to a Classic Condition

**DOI:** 10.3390/ijms21134690

**Published:** 2020-06-30

**Authors:** Laura Martinez Valenzuela, Juliana Draibe, Xavier Fulladosa, Juan Torras

**Affiliations:** 1 Bellvitge University Hospital, Nephrology Department, Hospitalet de Llobregat, 08907 Barcelona, Spain; lmartinezv@bellvitgehospital.cat (L.M.V.); jbordignon@bellvitgehospital.cat (J.D.); xfulladosa@bellvitgehospital.cat (X.F.); 2 IDIBELL Biomedical Research Institute, Hospitalet de Llobregat, 08907 Barcelona, Spain; 3 Clinical Sciences Department, Campus de Bellvitge, Barcelona University, Hospitalet de Llobregat, 08907 Barcelona, Spain

**Keywords:** acute tubulointerstitial nephritis, immunology, biomarkers

## Abstract

Acute tubulointerstitial nephritis (ATIN) is an immunomediated cause of acute kidney injury. The prevalence of ATIN among the causes of acute kidney injury (AKI) is not negligible, especially those cases related to certain drugs. To date, there is a lack of reliable non-invasive diagnostic and follow-up markers. The gold standard for diagnosis is kidney biopsy, which shows a pattern of tubulointerstitial leukocyte infiltrate. The urinalysis findings can aid in the diagnosis but are no longer considered sensitive or specific. Atthe present time, there is a rising attentiveness tofinding trustworthy biomarkers of the disease, with special focus in urinary cytokines and chemokines that may reflect kidney local inflammation. Cell-based tests are of notable interest to identify the exact drug involved in hypersensitivity reactions to drugs, manifesting as ATIN. Certain single-nucleotide polymorphisms in HLA or cytokine genes may confer susceptibility to the disease according to pathophysiological basis. In this review, we aim to critically examine and summarize the available evidence on this topic.

## 1. Introduction

Acute tubulointerstitial nephritis (ATIN) is an immunomediated disease affecting the tubulointerstitial area of the kidneys. The tubulointerstitium comprises 80% of the kidney surface and is composed of cellular and extracellular matrix components [1]. The specific and identifying pathological picture consists of a cellular infiltrate composed by leukocytes, primarily lymphocytes, but also including eosinophils, macrophages, or plasma cells. A myriad of etiologies can lead to ATIN, although drug-induced ATIN is the most common, accounting for 3–14% of biopsy-proven acute kidney injury (AKI) [2] and 70–90% of ATIN cases [3]. Also, of note, ATIN is related to autoimmune and inflammatory diseases—systemic lupus erythematosus, IgG-4 related disease, and tubulointerstitial nephritis with uveitis (TINU) syndrome, among others—or infectious diseases, such as cytomegalovirus or adenovirus infections. 

Type IV hypersensitivity reactions are the underlying pathomechanisms of drug-induced ATIN. The kidneys are adapted to filter a high rate of blood flow that contains proteins and potential antigens, thus leaving them exposed to drugs and their metabolites [4]. Tubular epithelial cells (TECs) are able to process and present these antigens from tubular lumen, working as non-professional antigen-presenting cells (APCs). TECs present antigens to dendritic cells, which in turn migrate to regional lymph nodes, activate specific T cells, and integrate innate and adaptive immune responses [5]. Those activated T cells infiltrate the renal parenchyma and amplify inflammation through increased secretion of cytokines and the recruitment of other inflammatory cells [4].

The involvement of necroinflammation pathways has been recently described to collaborate in the pathogenesis of drug induced ATIN. It has been hypothesized that drugs can directly damage TECs and induce necroptosis. This is a recently-described form of cell death, halfway between necrosis and apoptosis, leading to the release of proinflammatory cytokines and the recruitment of innate immune system cells. After necroptosis of TECs, intracellular molecules are dropped to the interstitial space and bind to several receptors that recognize danger signals, such as toll-like receptors (TLRs), expressed by immune cells. Signal–receptor interaction leads to the release of proinflammatory cytokines that, in turn, magnify the immune response, triggering further direct TEC necroptosis [6]. A role for necroinflammation has been confirmed in a murine model of cisplatin-induced AKI, but further research is required to confirm the participation of these pathways in human ATIN [7]. 

Inflammatory phenomena and cellular infiltration lead to tubular dysfunction and (AKI). It is usually difficult for the clinician to distinguish between ATIN and acute tubular necrosis (ATN) in this setting. ATIN may be accompanied by fever, skin rash, arthralgias, or flank pain, contrarily to ATN, and this picture can help guide the diagnosis, but those are not universal findings. The presence of known previous autoimmune conditions, concomitant infections, or recently-administered drugs can also support the hypothesis of ATIN of a specific etiology. The gold standard in ATIN diagnosis is kidney biopsy. Based on the predominance of an inflammatory component in the kidneys of ATIN, some classical and novel biomarkers, reviewed hereunder, may serve in the diagnosis, prognosis, and follow-up of this disease. 

## 2. ATIN Classical Biomarkers

Urine cellularity and casts have been used classically to find evidence of localized inflammation in the kidneys. Routine optical microscopy examination of the urine samples requires trained personnel and is time-consuming. In recent decades, automated cytometric urinalysis has replaced provider-performed urine microscopy. The information obtained here may guide the diagnosis but has limitations. Occasionally, urine sediment can be negative despite the existence of inflammatory kidney disease, and the presence of cells and crystals is not always specific to a certain pathology. Also, automated examination is less precise for diagnosis than laboratory-based microscopy examination. Thus, although useful, urine sediment examination should always be accompanied by knowledge of the clinical context [8].

Sterile leukocyturia is a common finding in ATIN patients. Depending on the series, the prevalence of leukocyturia ranges within 50–70% of all-cause-ATIN cases [9,10]. Interestingly, leukocyturia is an almost universal finding in drug-related and especially in antibiotic-related ATIN, while it is found merely in about 50% of ATIN patients related to autoimmune diseases [11]. 

Due to the Type IV hypersensitivity basis of drug-induced ATIN and the usual presence of eosinophils in kidney biopsy specimens [6]., eosinophiluria was considered a classical biomarker in ATIN. The belief inthe utility of this parameter is based on a small-case series. Although the increased sensitivity was noted using Hansel stain instead of Wright stain [12], eosinophiluria is no longer considered sensitive or specific. In the largest cohort studied, Muriithi et al. found 31% sensitivity and 68% specificity for the diagnosis of ATIN among 566 patients with AKI, and found no utility of eosinophiluria in the distinction between ATIN and ATN [13]. Patients with urinary tract abnormalities, urinary tract infections, acute tubular necrosis, and glomerulonephritis also exhibit eosinophiluria [14].

Other findings from urine microscopic examination are white blood cell (WBC) casts, which are either not sensitive and not specific for ATIN diagnosis. WBC casts are found in urine from patients with inflammatory kidney diseases, not only ATIN. They have also been seen in other conditions such as glomerulonephritis and pyelonephritis. Surprisingly, less than 14% of ATIN patients present WBC in urine microscopy according to the published series. Red blood cell (RBC) casts were once thought to be specific for glomerular disease, however up to one third of ATIN patients exhibit RBC, probably related to disruption of interstitial blood vessels and leakage of erythrocytes to the tubular lumen [15]. Granular casts are the most frequent casts seen in ATIN, up to 95% [16]. depending on the series, but they are also very common in ATN. 

## 3. Novel Biomarkers

ATIN involves cell immunity rather than humoralmechanisms, as suggested by the extensive and pleiomorphic inter-tubular cell infiltrate in ATIN kidney biopsies, with lack of immune deposits in most of the cases. Rarely anti-tubular basal membrane (TMB) antibodies and immune complexes can be noticed. The presence of this immune cell infiltration can damage TECs due to direct cytotoxicity or local cytokine release. At the same time, TECs acquire an active role in inflammation by orchestrating the immune response and directly producing diverse cytokines. In the same line, cell infiltrates together with activated TECs also produce signaling molecules that promote matrix deposition and remodeling, thus promoting fibrosis [17].

Identification of the differential nature of the tubulointerstitial infiltrates in ATIN and the cytokines produced by this infiltrate—in comparison to other inflammatory kidney diseases—and their detection in serum or urine, can be useful as biomarkers of the disease.

## 4. Serum and Urine Cytokines and Chemokines

In the recent years, there has been a rise in the number of studies published in the field of urinary biomarkers, seizing the non-invasive nature of the sampling. The introduction and spread of novel multiplex assays that allow multiple simultaneous cytokine measurements in the same procedure has become a valuable tool in this kind of research. The presence in urine of the different chemokines and cytokines evaluated isillustrative of the pathophysiologic processes occurring in this disease.

Monocyte chemoattractant protein-1 (MCP-1) has a key role in the recruitment of monocytes, neutrophils, and lymphocytes in tissue inflammation processes. In the kidney, it is produced by TECs, endothelial cells of the peritubular capillaries, and macrophages themselves. MCP-1 has been identified as a chemokine involved in autoimmune diseases [18]. Wu et al. found higher MCP-1 levels in a cohort of 40 patients with drug-induced ATIN compared to controls. Urinary MCP-1 correlated and predicted the severity of the acute lesions in kidney biopsies from these patients [19]. In the same line, Yun et al. described higher MCP-1 serum and urine concentration among 113 ATIN patients from different causes in their bead-based multiplex assay [20]. Interestingly, Dantas et al. found that urinary MCP-1 concentration finely correlated with the amount of tubulointerstitial infiltrate but not with the glomerular infiltrate in a cohort of patients with glomerular autoimmune diseases [21]. Other authors also reported higher urinary levels of MCP-1 in patients with systemic lupus erythematosus (SLE) that correlated with the extension of the tubulointerstitial infiltrate, thus confirming the utility of MCP-1 in urine as a biomarker of acute infiltration of kidneys with dense affectation of this compartment [22].

Tumor necrosis factor alpha (TNF-α) is a proinflammatory cytokine produced by macrophages and monocytes. It participates in signaling cascades that lead to cell apoptosis or necrosis. Several authors reported higher levels of TNF-α in serum and urine samples from ATIN patients. Moledina et al. reported a higher urinary TNF-α in patients with ATIN that helped in the differential diagnosis of patients with ATN [23]. The same group showed that urinary TNF-α was also higher in a large series of ATIN cases compared to other causes of inflammatory kidney disease, and that urinary TNF-α correlated well with the number of TNF-α-positive cells in the renal biopsy [24]. Aoyagi et al. reported the value of serum TNF-α levels in the follow-up of a case of TINU, which dramatically decreased during the first week after treatment initiation [25]. Moledina et al. found higher urinary IL-9 along with higher TNF-α levels in patients with ATIN. IL-9 is involved in allergic responses and induces mast cell accumulation, which isin turn a source of TNF-α [23,24]. TNF-α inducessecretion of IL-6, among other cytokines. This is a cytokine with a local proinflammatory effect in the early stages of inflammation that also induces acute-phase production of proteins such as CRP [26]. Numerous authors reported elevated IL-6 concentration in urine and plasma from patients with ATIN compared to healthy controls [20,27,28].

**Figure 1:**
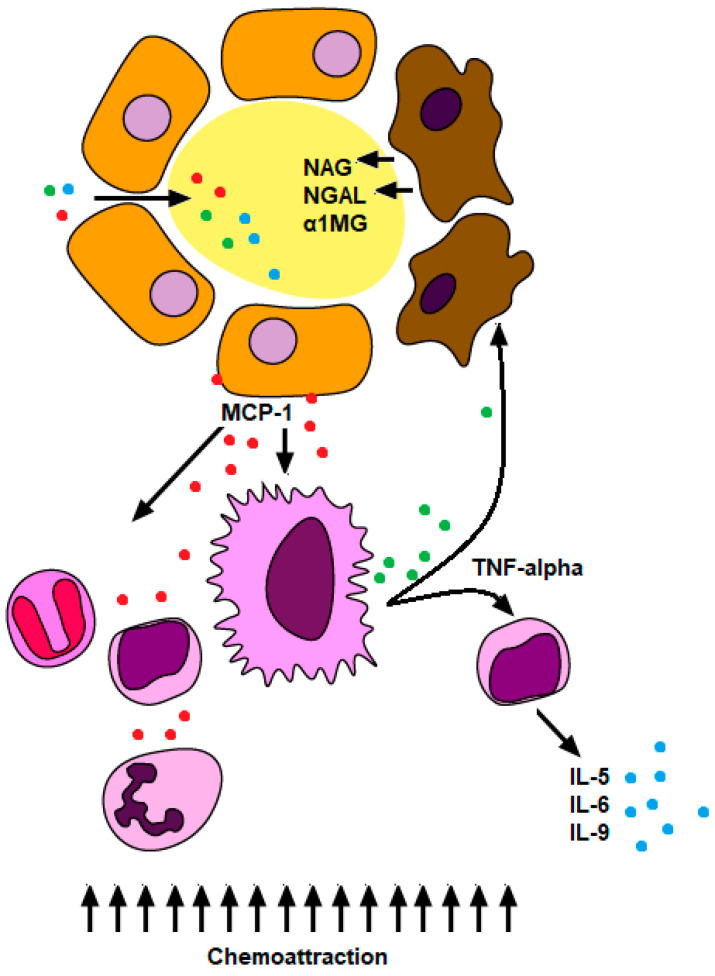
Cytokines and chemokines suggested as biomarkers in acute tubulointerstitial nephritis. Tubular endothelial cells (TECs) secrete MCP-1, which is a powerful chemoattractant for inflammatory cells towards the interstitium. Incoming macrophages release TNF-α, that primes and activates lymphocytes to secrete various proinflammatory interleukins (IL). In parallel, TNF-α can damage TECs inducing acute tubular necrosis (ATN). As a consequence of ATN, some markers of the injury of TECs (NAG, NGAL, and α1MG ) are found in urine.MCP-1, monocyte chemoattractant protein-1; TNFα, tumor necrosis factor α; NAG, N-acetyl glucosamidinadase; NGAL, neutrophil gelatinase-associated lipocalin; α1MG, α1 microglobulin.

Other unspecific tubular AKI biomarkers have also been evaluated in ATIN. *N*-acetyl-β-D-glucosaminidase (NAG) is a lysosomal enzyme present in TECs which is a marker of proximal renal tubular damage [29]. Neutrophil gelatinase-associated lipocalin (NGAL) is a protein contained in the neutrophil granules but also in other human tissues such as kidney TECs. Some authors have demonstrated NGAL release during inflammatory processes [30]. α1-microglobulin is a low-molecular-weight protein that acts as radical scavenger and reductase [31]. NAG, α1-microglobulin, and NGAL are filtered in the glomeruli and completely reabsorbed by proximal TECs. Thus, urinary presence of these three molecules indicates proximal TEC damage and dysfunction. Many authors have found high levels of these biomarkers in urine and have used combination strategies to increase sensitivity and specificity of ATIN diagnosis [19,32,33,34]. Figure 1 illustrates the role of the suggested cytokines and chemokines in ATIN.

Renal regional complement activation has been examined as an ATIN biomarker in a study by Zhao et al. Soluble urinary C5b9 was found to correlate with the degree of interstitial inflammatory infiltrates and also with tubular dysfunction in a cohort of 44 patients with ATIN of different etiologies [35].

In addition to the diagnostic value in the acute stage of the disease, urinary biomarkers have been evaluated in other settings. Shi et al. evaluated a panel of biomarkers in the follow-up of 54 patients with ATIN along a median time of 38 months. They found that NAG, matrix metalloproteinase 2 (MMP)2, and MMP9 clearly correlated with the rate of glomerular filtration rate (GFR) decline. The authors performed a multiple linear regression analysis to exclude other factors, and confirmed that higher concentration of these biomarkers at diagnostic was independently associated with faster progression of chronic kidney disease [34]. Table 1 summarizes the published evidence of ATIN serum and urinary cytokines and chemokines as biomarkers.

## 5. Cellular Biomarkers

In the context of drug-induced ATIN, the most common cause of this group of diseases, in vivo cellular assays are key to demonstrate the existence of drug hypersensitivity and identify the offending drug. Throughout their lives, but also during a previous ATIN episode, people are frequently exposed to multiple drugs. Proper identification of which exact medication is the culprit allows for safe discontinuation. Binding of T cells with a drug is a complex process that generates a cascade of events that can be measured in its different steps. These cellular assays are based on the demonstration of the lymphocyte proliferation response and the cytokine secretion response when exposed to the suspected drug.

On the one hand, the activation phenotype of lymphocytes after drug exposure can be assessed using fluorescence cytometry techniques. Detection of activation markers onthe surface of lymphocytes after incubation with the offending drug—such as CD25, CD69, or Human Leukocyte Antigen (HLA)-DR—may indicate hypersensitivity [36,37].

Also, activation of T cells after drug exposure triggers a specific pattern of cytokine secretion. These cytokines can be detected in the supernatant of the stimulated cell cultures using enzyme- linked assays. Many authors have reported the release of high amounts of IL-5 in the setting of hypersensitivity. Mauri-Hellweg et al. reported high IL-5 concentration in supernatant of lymphocyte cultures from patients with known hypersensitivity to diverse antiepileptic drugs after culturing with these drugs^37^. Zanni et al. found a predominant Th2 response with prominent IL-5 release among a cohort of 13 patients affected by lidocaine hypersensitivity [38]. Sachs et al. found higher IL-5 concentration and, to a lesser extent IL-10 and IFN-γ, was even more sensitive for the detection of drug hypersensitivity than lymphocyte proliferation tests [39].

Enzyme-linked immunospot (ELISpot) assay is a sensitive test that allows the ex vivo measurement of the production of cytokines by lymphocytes in response to a certain stimulus. It provides quantitative information of the amount of responder cells. There are some reports of the usefulness of this test in evaluating drug hypersensitivity reactions. Tanvarasethee et al. studied 25 patients with cephalosporin-induced maculopapular exanthema. They found that the combined quantification of INF-γ and IL-5 spots by ELISpot assay was more sensitive than skin tests to diagnose cephalosporin hypersensitivity [40]. Rozieres et al. proved the specificity of the IFN-γELISpot assay for the demonstration of Type IV delayed hypersensitivity reaction (DHR) detecting amoxicillin-specific T cells. Twenty of twenty-two patients with known amoxicillin DHR had detectable amoxicillin-specific T cells. Interestingly, none of the control patients with IgE-mediated amoxicillin hypersensitivity nor the healthy controls presented amoxicillin-specific T cells [41]. At the moment, there is little evidence on the usefulness of ELISpot assays specifically targeting patients with ATIN. Punrin et al., in a cohort of patients with drug-induced ATIN, reported a positive IFN-γELISpot assay in 50% of them [42].

The lymphocyte transformation test (LTT) measures the proliferation of lymphocytes in response to the pure form of a suspicious drug. In vitro, prior to the exposure, lymphocytes are incubated with ^3^H-thymidine or carboxyfluoresceinsuccinimidyl ester (CFSE). The attenuation of radioactivity incorporation or dilution of CFSE with each cell division after incubation with the offending drug, measured in a β-counter or flow cytometry, is an indicator of cell proliferation. Positive LTT has been reported in the setting of ATIN induced by β-lactams and NSAIDs [43]. Koda et al. described a case of ATIN in the setting of the treatment with nivolumab and lansoprazole [44]. The LTT demonstrated reactivity against lansoprazole and not against nivolumab, indicating which was the culprit drug for ATIN.

## 6. Genetic Biomarkers

Some descriptions of the association of ATIN, mainly in the setting of TINU, with certain human leukocyte antigen (HLA) or single-nucleotide polymorphisms (SNPs) as markers of disease susceptibility have been published.

To identify genetic markers of TINU, Levinson et al. performed HLA genotyping of 18 American TINU patients. They found that the disease was strongly associated with HLA-DQA1*01, HLA-DQB1*05, and HLA-DRB1*01. The prevalence of these HLA genes was elevated among their cohort of TINU patients, and significantly higher in comparison with the published HLA frequencies in North American whites [45]. A study conducted in a cohort of 154 Chinese patients with ATIN of different causes showed that, similarly to the American study, HLA-DQA1*0104/DQB1*0503/DRB1*1405 are risk haplotypes for the development of ATIN. These studies suggest that these variants may enhance antigen presentation and facilitate renal tubulointerstitial inflammation.

Rytkönen et al. studied IL-10 gene SNPs in a cohort of 30 pediatric cases of TINU or idiopathic TIN. They found a higher prevalence of the IL-10 SNP s3024490 in TINU/ATIN cases compared with its prevalence in a control group of 393 individuals from their own population [46]. IL-10 is an immunoregulatory cytokine that inhibits the synthesis of proinflammatory cytokines and chemokines. Interestingly, IL-10 SNPs have also been linked to susceptibility to recurrent attacks of acute pyelonephritis [47]. Based on these findings, the authors suggested that patients with SNPs in immunoregulatory cytokines and other inflammatory mediators may confer higher susceptibility to TINU/ATIN.

## 7. Final Remarks

To date, there is no reliable, non-invasive test for the diagnosis and monitoring of ATIN. Classical urine studies have shown poor sensitivity and specificity in ATIN diagnosis. Kidney biopsy is the gold standard for the assessment of ATIN, but there are significant drawbacks in the performance of this procedure in certain settings.First, it is contraindicated in certain patients such as those with uncontrolled hypertension or non-corrigible coagulation disorders, and the indication in the case of mononephric, small-sized kidney, or obese patients may be questionable due to the increased risk of serious complications.Second, although infrequent, variable degrees of bleeding may occur during the procedure or the following hours, especially when inflammation is prominent. Third, kidney biopsy requires trained personnel and a sufficient infrastructure that is not generally available in all hospitals, and a shift to specialized units may causedelays in the diagnosis.

Urinary biomarkers are excellent candidates for the evaluation of kidney diseases. Urine sampling has the advantage of being a non-invasive, immediate, and easy-to-performprocedure and can be repeated over time.The infrastructure required for cytokine quantification is relatively simple and frequently ELISA based, thus its use can be widespread.Urinary molecular content is a valuable live, and direct reflection of the inflammatory reactions occurring locally in the kidney tissue.The selection of novel biomarkers based on the pathophysiology of the disease will, over time, permit refinement in the diagnosis of the disease and the follow-up of the immune activity.

Cell-based assayshave an added value because they go beyond the diagnosis of ATIN. They confirm the presence of a DHRagainst aspecific drug in ATIN patients. Genetic biomarkers can help to deepen the knowledge of the pathophysiology of the disease.

Summarizing, novel biomarkers of ATIN are of interest to avoid kidney biopsy and its complications, are based on non-invasive sampling techniques, are low-resource consuming, reflect pathogenic processes ongoing in the kidney tissue, and can help identify the culprit drug in the case of drug-induced ATIN.

## Figures and Tables

**Table 1:** Summary of the main publications related to serum and urinary biomarkers of acute tubulointerstitial nephritis (ATIN).

Reference	Population Samples	RelevantFindings
Dantas et al., Kidney Blood Press Res, 2007	Glomerulopathy *n*=37	Urinary MCP-1 correlated with the extent of tubulointerstitial infiltrate by macrophages but not with the degree of glomerular infiltrate.
Yu et al., Journal of Peking University, 2010	Acute drug-induced TIN *n*= 28 Chronic drug-induced TIN *n*=12	The combination of urinary NAG and α1-MG increased sensitivity and specificity for the detection of acute drug-induced tubulointerstitial nephritis (TIN).
Wu et al., Clin J Am SocNephrol, 2010	Drug-induced ATIN *n*=40 Healthy controls *n*=20	MCP-1, α1-MG, NGAL, and NAG urinary levels were higher in ATIN patients compared to controls. MCP-1 urinary levels correlated with the extent and severity of the acute lesions.
Nakashima et al., ClinNephrol, 2010	IgG 4 disease-related ATIN *n*=4 Other cause ATIN *n*=16	IL-4, IL-10, and TGFβ RNA expression in kidney tissue was higher in IgG4 disease related ATIN compared to the rest of ATIN causes.
Shi et al., Am J Med Sci, 2013	Drug-induced ATIN *n*=51	Patients with higher urinary levelsof NAG, metalloproteinase 2(MMP2) and MMP9 presented faster GFR decline during follow-up
Wu et al., Am J Med Sci, 2013	ATIN *n*=40 Healthy controls *n*=20	Urinary α1-MG correlated with the degree of interstitial edema and inflammatory infiltrate in kidney biopsy. Urinary NAG correlated with the degree of inflammatory infiltrate. Urinary TGFβ correlated with the presence of fibrosis.
Aoyagi et al., CEN Case Rep, 2014	One case of TINU	Serum TNFα, IL-8 and IFNγ levels decreased during follow up of an episode of TINU.
Chen et al. Braz J Med Biol Res, 2018	ATIN *n*=30 Healthy controls *n*=15	Serum IL-6, IL-10, and TNFαwere significantly higher in ATIN patients compared to controls.
Zhao et al., Am J Physiol Renal Physiol, 2019	ATIN *n*=44 Healthy controls *n*=24	Urinary levels of KIM-1 and C5b9 were higher in ATIN patients compared to healthy controls. Urinary C5b9 correlated with the extent of tubulointerstitial infiltrates in kidney biopsy in ATIN patients.
Yun et al., BMC nephrology, 2019	ATIN *n*=113 Healthy controls *n*=40	Serum IL-1β, IFNα2, TNFα, MCP-1, IL-8, IL-17A, IL-18, and IL-23 were higher in ATIN patients compared to healthy controls. Urinary IFNα2, MCP-1, IL-6, IL-8, IL-12p70, and IL-17A were higher in ATIN patients compared to healthy controls
Moledina et al., JCI Insight, 2019	ATIN *n*=32 Other kidney diseases *n*=186	Urinary TNFα and IL-9 were higher in ATIN patients compared to other kidney diseases. Urinary IL-5 was higher among those ATIN patients with prominent eosinophil infiltrates.
Moledina et al., Nephron, 2019	ATIN *n*=32 ATN *n*= 41	Urinary TNFα and IL-9 were higher in ATIN patients.

MCP-1 macrophage chemoattractant protein; NAG N-acetyl-neuraminidase; α1-MG α1-microglobulin; NGAL neutrophil gelatinase-associated lipocalin; IL-4 interleukin-4, IL-10 interleukin-10, transforming growth factor-β; GFR glomerular filtration rate; TNFα Tumor Necrosis Factor α; IL-8 Interleukin-8; IFNγ interferon γ; IL-6 interleukin-6; KIM-1 kidney injury molecule; IL-1β interleukin 1 β, IFNα2 interferon α2; IL-17A interleukin-17A, IL-18 interleukin-18; IL-23 interleukin-23; IL-12p70 interleukin-12p70; IL-9 interleukin-9; IL-5 interleukin-5.
